# Icariin protects rats against 5/6 nephrectomy-induced chronic kidney failure by increasing the number of renal stem cells

**DOI:** 10.1186/s12906-015-0909-8

**Published:** 2015-10-21

**Authors:** Zhongdi Huang, Liqun He, Di Huang, Shi Lei, Jiandong Gao

**Affiliations:** Department of Hematology, Shuguang Hospital Affiliated to Shanghai University of Traditional Chinese Medicine, 185 Pu’an Road, Shanghai, 200021 China; Department of nephrology, Shuguang Hospital Affiliated to Shanghai University of Traditional Chinese Medicine, 528 ZhangHeng Road, Shanghai, 201203 China; Traditional Chinese Medicine Institute of Kidney Diseases, Shanghai University of Traditional Chinese Medicine, 528 ZhangHeng Road, Shanghai, 201203 China; Shanghai Key Laboratory of Traditional Chinese Clinical Medicine, 528 ZhangHeng Road, Shanghai, 201203 China; Collage of Biology and pharmacy, China Three Gorges University, 8 Daxue Road, Yichang, Hubei 443002 China

**Keywords:** Chronic kidney failure, Icariin, Renal function, Renal stem cells

## Abstract

**Background:**

Chronic kidney disease poses a serious health problem worldwide with increasing prevalence and lack of effective treatment. This study aimed to investigate the mechanism of icariin in alleviating chronic renal failure induced by 5/6 nephrectomy in rats.

**Methods:**

The chronic renal failure model was established by a two-phased 5/6 nephrectomy procedure. The model rats were given daily doses of water or icariin for 8 weeks. The kidney morphology was checked by HE staining. The levels of blood urea nitrogen, serum creatinine, and serum uric acid were measured by colometric methods. The expression of specified genes was analyzed by quantitative real-time PCR and immunohistochemical staining. The number of renal stem/progenitor cells was analyzed by CD133 and CD24 immunohistochemical staining.

**Results:**

Icariin protected against CDK-caused damages to kidney histology and improved renal function, significantly reduced levels of BUN, creatinine, and uric acid. Icariin inhibited the expression level of TGF-β1 whereas upregulated HGF, BMP-7, WT-1, and Pax2 expression. Moreover, ccariin significantly increased the expression of CD24, CD133, Osr1, and Nanog in remnant kidney and the numbers of CD133^+^/CD24^+^ renal stem/progenitor cells.

**Conclusions:**

These data demonstrated that icariin effectively alleviated 5/6 nephrectomy induced chronic renal failure through increasing renal stem/progenitor cells.

## Background

The kidneys function as the cleansing and recycling apparatus of the body removing waste products of metabolism and water soluble wastes from blood, excreting urea and ammonium, reabsorbing water, glucose, and amino acids, by which they serve homeostatic functions such as the regulation of electrolytes, maintenance of acid-base and salt-water balances, and regulation of blood pressure. Besides, the kidneys also serve as an endocrine organ producing hormones calcitriol and erythropoietin among others, and the enzyme rennin. Chronic kidney disease (CKD) is a collective term for any process causing the pathological changes to the structure of kidney which may consequently reduce renal function and disturbs the physiological homeostasis, a process collectively termed [1, 2]. There is a continuous increase of the prevalence of CDK worldwide [3–5], which results in dramatic increasing incidences of end-stage renal disease (ESRD) [2] that require renal replacement therapy or undergoing dialysis due to lack of kidney donor.

Stem cell research and regenerative medicine provide a hope for treating CKD and even ERSD. However, it is extremely challenging to reconstruct human kidney because of its complicated anatomical structure and no regenerating zone of renal tissue to form new nephrons [6]. A variety insults could injure different renal cells including podocytes, tubular epithelial cells, mesangial cells, and endothelial cells. While the sublethal injuries damage renal function to different degrees, they could also activate the regenerating process for repairing the injuried kidney tissues [7]. It has been shown that renal dysfunction could be reversed by stimulating the angiogenic signaling and increasing renal microvasculature density [8, 9]. Many animal studies have shown that renal cells could be modulated genetically or pharmacologically to promote kidney regeneration [3, 10].

Icariin, a flavonoid from plants of genus *Epimedium*, has been shown therapeutic potential for neurodegeneration, memory and depressive disorders, chronic inflammation, cardiovascular diseases, diabetes, osteoporosis, cancer, reproductive disorders, and immune dysfunction [11, 12]. Icariin promoted self-renewal of mouse neural stem cells in vitro [13], the activation of quiescent neural stem cells of aged rats in vivo [14], and the differentiation of mesenchymal or stem cells into cardiomyocytes, osteoblasts, and endothelial cells [15–18]. The aim of this study was to investigate whether icariin could modulate renal stem cell population to repair kidney injury and alleviate chronic renal failure in a rat 5/6 nephrectomy model.

## Methods

### Animals

Eighty male Sprague Dawley rats (SPF class, 150 ± 10 g) were purchased from Shanghai Laboratory Animal Research Center (Shanghai, China) and kept in the laboratory animal center of Shanghai University of Traditional Chinese Medicine. The animals were given food and water *ad libitho*. All animal procedures were adhered to the Declaration of Helsinki and approved by the IACUC of Shanghai University of Traditional Chinese Medicine (SCXK (Shanghai) 2008-0016).

The renal failure rat model was established by a two-phased procedure of 5/6 nephrectomy. Rats were given 3 % sodium pentobarbital at 2 ml/kg dose intraperitoneally. After anesthesia, rats were put into left lateral position, shaved, disinfected with 75 % ethanol. Locating the kidney under rib ridge and cutting a about 2 cm opening on skin, then cut the muscles; use tweezers to pull up the kidney out of surrounding fat, peel off renal cell membrane from the lower end (to avoid injury to adrenal gland); cut off about 5/6 renal tissue near both ends but keep parts around the renal hilum to avoid disruption of blood supply; wrap remaining kidney twice with a thick line and stretch 10 min with hemostat. When there was no further bleeding, the opening was sutured layer by layer and wiped clean with cotton swab. A week later the right renal hilum was ligated with thick lines to make kidney necrosis. The sham group rats were undergone the same procedure but without cutting any kidney tissue. The establishment of renal failure model was confirmed by serum creatinine level 2 weeks later.

### Treatments

The rats were randomly put into sham, CKD model, and groups with 20 rats in each group. Sham and control rats were given water 3 ml/day, icariin group were given icariin (40 mg/kg/d, CAS:489-32-7, Shanghai Winherb Medical Technology, Shanghai, China) in 3 ml/day by gavage for 8 weeks. Then the rats were sacrificed by approved standard procedure, blood was collected via celiac artery, and kidney was divided into two sections longitudinally, one part for RNA work and the other for hematoxylin and eosin (H&E) and immunohistochemical staining.

### Kidney function assays

Serum urea nitrogen was measured by diacetyloxime colorimetric method, serum creatinine by picric acid method, and uric acid by colorimetric method.

### Kidney histology

Kidney tissue was fixed in 10 % formalin and sectioned followed by Hematoxylin and Eosin (H&E) staining and light microscopy (Olympus IX70, Olympus, Shinjuku, Tokyo, Japan) observation.

### Reverse transcription and quantitative polymerase chain reaction (qPCR)

Total mRNA was extracted from kidney tissue samples using Trizol (Life Technologies, Shanghai, China) method. The first strand cDNA was synthesized with 2 μg total RNA using a reverse transcriptase kit from Biotnt (Shanghai, China) according to manufacturer’s protocol. Quantitative PCR was performed in 20 μl total reaction mix using Fast SYBR® Green Master Mix (Life Technologies) on a ABI 7500 fast (Applied Biosystems, Foster City, CA) with 95 °C for 5 min followed by 40 cycles of 95 °C 5 s and 60 °C 30 s. The sequences of specific primers were listed in Table 1. The relative gene expression levels were calculated by 2^-ΔΔCt^ method with β-actin as the internal control.

**Table 1:** Sequences of qPCR primers used in this study

Gene	Primer sequences	Amplicon (bp)
TGF-β1	GAAGGACCTGGGTTGGAAG	136
	CGGGTTGTGTTGGTTGTAG	
HGF	CCTATTTCCCGTTGTGAAG	138
	ACTAACCATCCACCCTACT	
BMP-7	ACTACTGTGAGGGAGAGTG	97
	TCTGGGTTGATGAAGTGAA	
Wt1	AAAAGTGGCTCACAGTGTC	139
	ATGGAACAACCGCTCTAAT	
Pax-2	CTGGGCAGGTACTACGAGA	190
	CGCTGGGAACTGTATCATT	
CD24	CCAGCCACCCCTGAGTAAATC	90
	GAACTTAGTACCCGTGGTGAGTGA	
CD133	ACGGAAGTCAGCTCCCATCA	76
	GGCTCTCCAGATCGGTTCTG	
Osr1	TTCTAAAGTGCCAGGTGCGG	79
	GACGTGTGGAAACCAGGGAA	
Nanog	TGCTCCGCTCCATAACTTCG	100
	AGTGGCTTCCAAATTCGCCT	
β-actin	CTCACTGTCCACCTTCCAGC	121
	AAGGGTGTAAAACGCAGCTCA	

### Immunohistochemical (IHC) staining

To detect the protein levels of TGF-β1, HGF, and BMP-7 and CD133 and CD24 positive cells in kidney tissue, IHC staining with specific antibodies was performed. The sections were deparaffinized and rehydrated, quenched with 3 % H_2_O_2_ for 10 min, immersed in citrate buffer and heated in microwave at mid power for 3 min, cooled down to room temperature and repeated heating another time before being washed twice in PBS at 5 min each and blocked with normal rabbit serum for 30 min at 37 °C, followed by incubating with specified 1st antibodies at 4 °C over night and proper 2nd antibodies at 37 °C for 30 min. The antibodies used were TGF-β1, HGF, and BMP-7 from Abcam (Cambridge, MA), CD24 from Santa Cruz (Santa Cruz, CA), and CD133 from Boster (Wuhan, China). The secondary antibodies were purchased from Jackson ImmunoResearch Lab (West Grove, PA). The sections were washed 3 times with PBS before color development with a DAB kit (SA1020, Boster, Wuhan, China) and counterstained with hematoxylin, washed, dehydrated, cleared, and mounted. The slides were observed and photographed on a Olympus IX70 (Olympus, Shinjuku, Tokyo, Japan). The immunohistochemical staining results were semi quantitatively analyzed by combining the score of staining intensity and the score of percentage of positive cells. The positive staining ranged from pale yellow to brown. Staining intensity was scored as 0 for now color, 1 for pale yellow, 2 for yellow, 3 for brown. The percentage of positive cells was scored as 0 for 0 to 5 %, 1 for 6 to 25 %, 2 for 26 to 50 %, 3 for 51 to 75 %, and 4 for >75 %. The final score was the product of the scores of staining intensity and the score of percentage of positive cells. Scores of 5 random fields (x400) were obtained from each slide.

### Statistical analyses

Data was expressed as mean ± standard deviation. Statistical analysis was performed using SPSS17.0 software package (IBM, Armonk, NY). The difference between the averages of groups was analyzed by one way ANOVA (analysis of variation), the homogeneity of variance between groups was determined by Levene’s test and set 0.10 significance level; multiple comparisons between two groups were performed by SNK test if variances were assumed equal and by Tamhane’s T2 test if variances were not assumed equal. A p value less than 0.05 was considered statistically significant.

## Results

### Icariin (Icariin) alleviated the pathological changes of chronic kidney disease

**Fig. 1:**
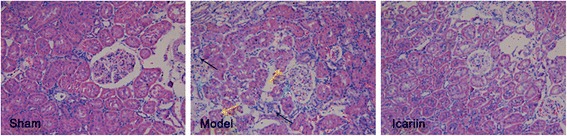
Icariin alleviated chronic renal failure induced morphological damages to rat kidney. The kidney histology of rats underwent sham operation, 5/6 nephrectomy (CKD model), and CKD treated with icariin was observed by Hematoxylin and Eosin staining. The representative pictures were shown. Yellow arrows indicated tubular lesion and tubules lacking obvious brush border; black arrows indicated tubular necrosis and inflammatory cell infiltration; and green arrows indicated glomerular damages

Hematoxylin and Eosin (H&E) staining showed that kidney morphology was well preserved in sham operated rats (Fig. 1a). It showed normal glomerular structure, capillaries were not narrowed or occluded, capillary basement membrane lined well without enlargement; Bowman’s capsule space was clear without expansion, glomerular capsule and glomerulus were separated; tubule structure was normal without protein cast or interstitial inflammatory cell infiltration.

Massive histological changes were observed in the kidneys of rats undergone 5/6 nephrectomy (model group) (Fig. 1b). The glomerular structure was disorganized with severe mesangial expansion, significant basement membrane thickening, and capillary compression or occlusion. Glomeruli had diffuse glomerular sclerosis with increased mesangial matrix area, reduced number of cells, glomerular fibrosis, and severe adhesions to Bowman’s capsules. A considerable amount of protein casts were observed in renal tubule lumen, which was significantly expanded with fibrosis and narrowing in some tubules. There was renal interstitial fibrosis and edema with large number of infiltrated inflammatory cells. Some small arteries showed intimal thickening, hyaline degeneration, and stenosis.

Compared to the model group, rats undergone 5/6 nephrectomy and received icariin treatment (Fig. 1c) had significantly reduced glomerular lesions with mild mesangial hyperplasia, mild basement membrane thickening, and unobvious glomerular sclerosis. Bowman’s capsules had normal morphology without significant adhesions with glomeruli. The structure of renal tubules was nearly normal with mild renal tubular epithelial cell swelling, lumen expansion, mild stenosis without apparent protein casts, and few interstitial inflammatory cell infiltration.

### Icariin protected renal function against chronic kidney disease

**Fig. 2:**
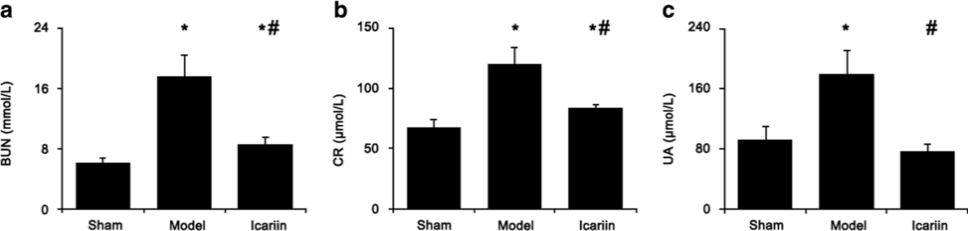
Icariin protected renal function against chronic kidney disease. The levels of blood urea nitrogen (**a**), serum creatinine (**b**), and serum uric acid (**c**) were measured by specific colometric methods. BNU, blood urea nitrogen; CR, serum creatinine; UA, uric acid. Data was expressed as mean ± standard deviation (*n* = 15). *, *p* < 0.01 compared to sham group; #, *p* < 0.01 compared to CKD model group

The blood urea nitrogen (BUN) level was increased from 6.084 ± 0.574 mmol/L (95 % CI: 5.371 – 6.797 mmol/L) in sham rats to 17.671 ± 2.820 mmol/L (95 % CI: 15.653 – 19.688 mmol/L) in CKD model rats (*p* < 0.01). This increased level of BUN in CKD rats was significantly reduced by icariin (Fig. 2a). The levels of serum creatinine (Fig. 2b) and uric acid (Fig. 2c) had similar changes as BUN in CKD rats among different treatment groups.

### Icariin promoted the expression of genes related to stem cell proliferation

**Fig. 3:**
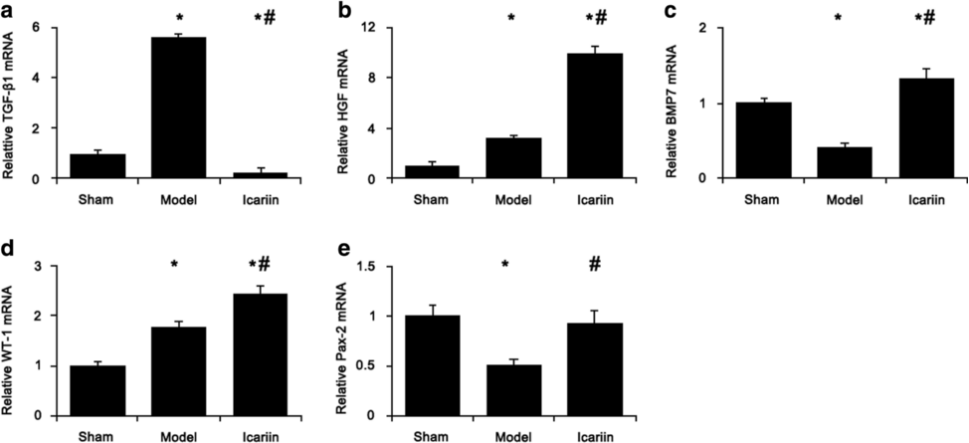
Icariin modulated kidney gene expression profile to favor injury repair. The mRNA levels of TGF-β1 (**a**), HGF (**b**), BMP-7 (**c**), WT-1 (**d**), and Pax-2 (**e**) were analyzed by quantitative real-time PCR. Data was expressed as mean ± standard deviation (*n* = 15). *, *p* < 0.01 compared to sham group; #, *p* < 0.01 compared to CKD model group

**Fig. 4:**
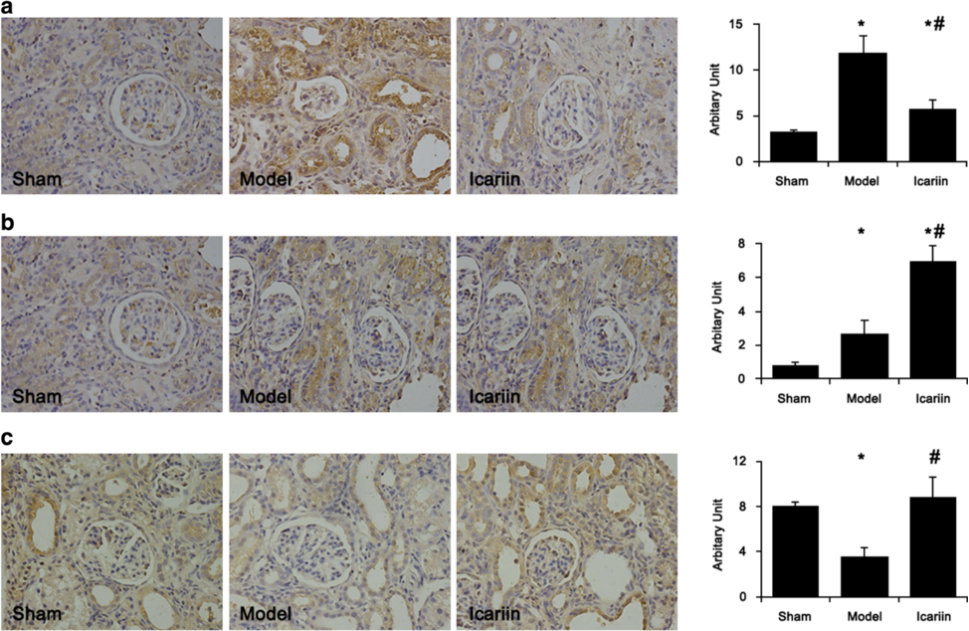
Icariin increased the protein levels of HGF and BMP-7 while reduced TGF-β1 level. The protein levels of TGF-β1 (**a**), HGF (**b**), and BMP-7 (**c**) in rat kidney were assayed by immunohistochemical staining with specific antibodies. Representative pictures and the quantitative summaries were given. *, *p* < 0.01 compared to sham group; #, *p* < 0.01 compared to CKD model group

Chronic kidney disease and icariin treatment each triggered changes of the expression of genes involved in stem cell/renal progenitor cell proliferation (Figs. 3 and 4). The renal mRNA levels of TGF-β1 (Fig. 3a), HGF (Fig. 3b), and WT-1 (Fig. 3d) were significantly higher whereas renal mRNA levels of BMP-7 (Fig. 3c) and Pax-2 (Fig. 3e) were significantly lower in CKD rats compared with those of healthy rats. Icariin drastically reduced the mRNA level of TGF-β1 (Fig. 3a) while increased the levels of HGF (Fig. 3b), BMP-7 (Fig. 3c), WT-1 (Fig. 3d), and Pax-2 (Fig. 3e) in CKD rats.

Consistently, the protein level of TGF-β1 was strongly elevated by injury-induced CKD, which was markedly inhibited by icariin and to a lesser extent by losartan (Fig. 4a). The HGF protein level in the residual kidney of CKD rats was significantly increased by icariin (Fig. 4b) while BMP-7 protein level in CKD rats was only recovered back to about the normal rats by icariin from more than 50 % decrease in CKD rats (Fig. 4c).

### Icariin promoted the proliferation of renal stem/progenitor cells

**Fig. 5:**
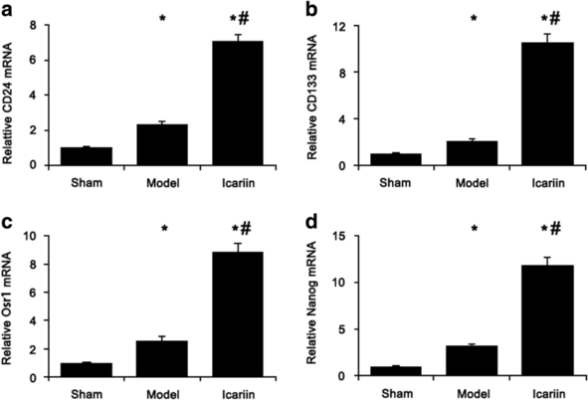
Icariin up-regulated the expression of renal stem cell markers. The mRNA levels of CD133 (**a**), CD24 (**b**), Osr1 (**c**), and Nanog (**d**) in the remnant kidney were analyzed by quantitative real-time PCR. Data was expressed as mean ± standard deviation (*n* = 15). *, *p* < 0.01 compared to sham group; #, *p* < 0.01 compared to CKD model group

The mRNA levels of markers of renal stem cell CD133 (Fig. 5a), CD24 (Fig. 5b), and Osr1 (Fig. 5c) and pluropotency (Nanog, Fig. 5d) were increased in the remnant kidney of 5/6 nephrectomy CKD rats and further increased 3-5 folds by icariin treatment (Fig. 5).

**Fig. 6:**
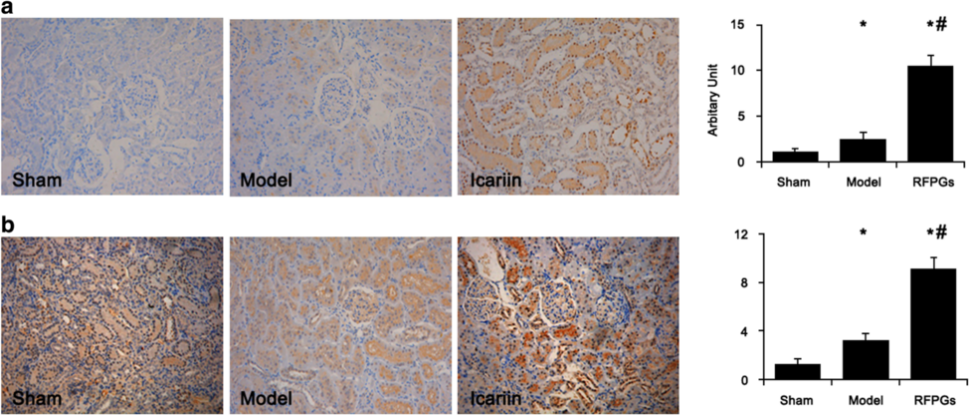
Icariin increased the numbers of CD133^+^ and CD24^+^ renal stem/progenitor cells. The numbers of CD133^+^ (**a**) and CD24^+^ (**b**) cells in rat kidney were semi-quantitatively measured by immunohistochemical staining with specific antibodies against CD133 and CD24. Representative pictures and the quantitative summaries were given. *, *p* < 0.01 compared to sham group; #, *p* < 0.01 compared to CKD model group

The number of CD133^+^ renal cells decreased about 65 % in CKD rats compared to healthy rats and icariin treatment resulted in about 5 fold increase of CD133^+^ cells in the kidney of CKD rats (Fig. 6a). The numbers of CD24+ cells had similar changes among healthy rats, CKD rats, and icariin or losartan- treated rats (Fig. 6b). Meanwhile, the numbers of renal stem/progenitor cells were correlated with kidney functions (Table 2), especially, CD133^+^ cell number showed tight negative correlation with BUN and creatinine levels.

**Table 2:** Correlation between renal function and the number of renal stem/progenitor cells

		BUN	Cr	CD133	CD24
BUN	Pearson Correlation	1	0.966**	−0.979**	−0.912*
	Sig. (1-tailed)		0.004	0.002	0.015
Cr	Pearson Correlation	0.966**	1	−0.946**	−0.799
	Sig. (1-tailed)	0.004		0.008	0.053
CD133	Pearson Correlation	−0.979**	−0.946**	1	0.935**
	Sig. (1-tailed)	0.002	0.008		0.01
CD24	Pearson Correlation	−0.912*	−0.799	0.935**	1
	Sig. (1-tailed)	0.015	0.053	0.01	

*BUN* blood urea nitrogen; *Cr* serum creatinine

*, *p* < 0.05; **, *p* < 0.01

## Discussions

Injury-induced chronic kidney disease caused profound histological changes including glomeruli damages, tubular necrosis, and infiltration of inflammatory cells in rat kidneys, which was accompanied with loss of renal functions indicated by the increase of BUN, creatinine, and uric acid levels. Icariin alleviated all such pathological changes, e. g. seldom tubular damages and much less immune cell infiltration, complete glomeruli structure, and nearly normal levels of BUN, creatinine, and uric acid in CKD rats received icariin. Furthermore, icariin significantly increased the numbers of CD133^+^ and CD24^+^ renal stem/progenitor cells in injury-induced CKD rats. Icariin activated the expression of genes promoting renal stem/progenitor cells proliferation and inhibited the expression of fibrosis-promoter TGF-β1.

**Fig. 7:**
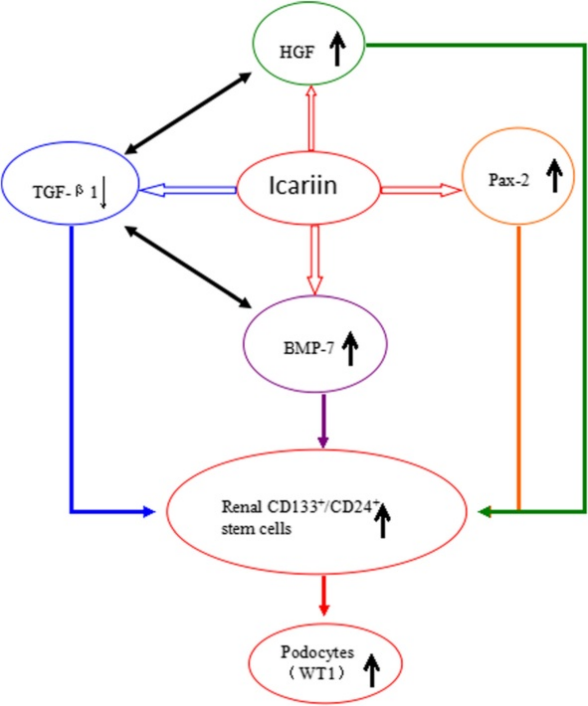
Proposed model for the actions of icariin in alleviating CKD. Icariin activates the expression of genes promoting the proliferation, survival, and differentiation of renal stem cells (e. g. HGF, BMP-7, Pax-2, and WT-1) and inhibits genes promoting inflammation and fibrosis (e. g. TGF-β1), which leads to the increase of renal stem/progenitor cells and the repair of kidney injuries

Renal regeneration is at the center of treating chronic kidney failure and other kidney diseases [10, 19, 20]. As icariin was renal protective by changing cell cycle distribution of renal cells [21] and was shown to promote the maintenance, activation, and differentiation of many types of stem cells [13–18], we postulated that the action of icariin in improving renal function would involve the activation of pathways promoting the proliferation and/or homing of renal stem/progenitor cells (Fig. 7). Among the factors involved in kidney development and kidney injury recovery, hepatocyte growth factor (HGF) and bone morphogenetic protein-7 (BMP-7) were analyzed and both found significantly increased by icariin in CKD rat kidney tissues. HGF/HGF receptor c-met signaling conferred renal protective effect by preventing apoptosis, reducing inflammation, and inhibiting TGF-β-induced fibrosis [22, 23]. BMP-7 promoted kidney regeneration by inhibiting the precocious differentiation of the kidney progenitor cells and antagonizing the TGF-β in inducing fibrosis [24, 25]. Moreover, icariin significantly up-regulated the expression of genes important for the proliferation and differentiation of renal stem/progenitor cells (Osr1, NMP-7, Pax2 and WT-1), which led to the increase of renal CD133^+^ and/or CD24^+^ stem/progenitor cells. Renal CD133^+^CD24^+^PDX^-^ cells were shown to be the uncommitted stem cells which possessed the potentials of self renewal and differentiation into both podocytes and tubular cells [26, 27]. The elevation of Osr1, Nanog, HGF, BMP-7, WT-1, and Pax2 expression in CKD rat kidney by Icariin was well in correlation with the increased numbers of CD133^+^ and/or CD24^+^ stem/progenitor cells while the reduced expression of TGF-β1 leading to the blocking of interstitial fibrosis (Fig. 7).

## Conclusions

In summary, icariin attenuated the progression of sub-total nephrectomy induced chronic renal failure in rats. Icariin moderated the loss of the integrity of kidney histology and renal function in CKD rats. Those structural and functional benefits of icariin were accompanied by the increased expression of genes promoting the proliferation of renal stem/progenitor cells as well as the increase of the numbers of CD133^+^ and/or CD24^+^ renal stem/progenitor cells.

## Abbreviations

CKD: Chronic kidney disease
ESRD: End-stage renal disease
H&E: Hematoxylin and Eosin
qPCR: Quantitative polymerase chain reaction
IHC: Immunohistochemistry
BUN: Blood urea nitrogen
